# Response to letter regarding “integrating systemic inflammation and liver biomarkers:prognostic implications of the ferritin index in heart failure”

**DOI:** 10.1080/07853890.2025.2568724

**Published:** 2025-10-07

**Authors:** Ching-Hui Huang, Chew-Teng Kor, Chia-Chu Chang

**Affiliations:** ^a^ Division of Cardiology, Department of Internal Medicine, Changhua Christian Hospital, Changhua, Taiwan; ^b^ Department of Mathematics, National Changhua University of Education, Changhua, Taiwan; ^c^ Department of Beauty Science and Graduate Institute of Beauty Science Technology, Chienkuo Technology University, Changhua, Taiwan; ^d^ Graduate Institute of Clinical Medicine, College of Medicine, National Chung Hsing University, Taichung, Taiwan; ^e^ Big Data Center, Changhua Christian Hospital, Changhua, Taiwan; ^f^ Graduate Institute of Statistics and Information Science, National Changhua University of Education, Changhua, Taiwan; ^g^ Department of Internal Medicine, Kuang Tien General Hospital, Taichung, Taiwan; ^h^ Department of Nutrition, Hungkuang University, Taichung, Taiwan

Dear Editors

We sincerely thank Xiaobo Shen et al. for their thoughtful and constructive comments on our article, *“Integrating systemic inflammation and liver biomarkers: prognostic implications of the ferritin index in heart failure”* [1]. Their insights allow us to clarify and expand on several points.

The ferritin index, as we presented, should be regarded as a hypothesis-generating biomarker. Its standalone performance was modest, but we believe it has potential value as part of a multi-marker approach. Our aim was not to propose it as a definitive predictor, but rather as a marker reflecting the interplay of inflammation, metabolism, and hepatic function in heart failure. By adjusting for age and sex, we sought to highlight its prognostic relevance beyond raw ferritin values, which have been the focus of prior studies. Given its limited discriminative power, the index may be most useful when combined with established predictors such as NT-proBNP.

We also recognize that the exclusion of patients without ferritin testing introduces selection bias, a reflection of the real-world underuse of iron studies in heart failure care. To address this, we compared included and excluded cohorts in detail (Supplementary Table 1), and while differences were observed, these underscore the unmet need for more routine ferritin assessment in clinical practice.

Another point of discussion concerns the inclusion of new-onset atrial fibrillation as part of our composite endpoint. We acknowledge that it differs mechanistically from events such as myocardial infarction or stroke. Nonetheless, atrial fibrillation is increasingly recognized as a major prognostic event in heart failure, strongly associated with hospitalization and adverse outcomes. Importantly, our sensitivity analysis excluding atrial fibrillation yielded consistent results, supporting the robustness of our findings regardless of endpoint definition (Supplementary Table 5).

We further acknowledge the limitation of relying on baseline ferritin alone, which does not capture chronic inflammatory activity. This constraint reflects the retrospective design of our study, and we agree that future prospective work should incorporate serial measurements. Similarly, ferritin alone cannot fully distinguish between inflammation and iron overload. To address this, we conducted subgroup analyses stratified by transferrin saturation, serum iron, C-reactive protein and NT-proBNP, which confirmed the robustness of the associations (Supplementary Table 3 and Supplementary Table 4).

Finally, as with any single-centre retrospective study, our findings are subject to inherent limitations despite adjustments such as IPTW. We therefore agree that multicentre, prospective studies integrating serial and complementary biomarkers will be essential to validate the clinical utility of the ferritin index.

In summary, we are grateful for these constructive remarks. We agree that the ferritin index should not be viewed as a definitive predictor, but rather as a marker of multisystemic inflammatory and metabolic stress in heart failure. Our results highlight its potential role in multi-marker strategies and emphasize the need for further prospective, multicentre validation with longitudinal data.

## Supplementary Material

Supplemental Material

## Data Availability

Data sharing is not applicable to this article as no data were created or analysed in this study.
